# Intuitive intellectual property law: A nationally-representative test of the plagiarism fallacy

**DOI:** 10.1371/journal.pone.0184315

**Published:** 2017-09-01

**Authors:** Anne A. Fast, Kristina R. Olson, Gregory N. Mandel

**Affiliations:** 1 Department of Psychology, University of Washington, Seattle, Washington, United States of America; 2 Beasley School of Law, Temple University, Philadelphia, Pennsylvania, United States of America; Northwestern University, UNITED STATES

## Abstract

Studies with convenience samples have suggested that the lay public’s conception of intellectual property laws, including how the laws should regulate and why they should exist, are largely incommensurate with the actual intended purpose of intellectual property laws and their history in the United States. In this paper, we test whether these findings generalize to a more diverse and representative sample. The major findings from past work were replicated in the current study. When presented with several potential reasons for IP protection, the lay public endorsed plagiarism and felt that acknowledging the original source of a creative work should make copying that work permissible—viewpoints strongly divergent from lawmakers’ intent and the law itself. In addition, we replicate the finding that lay people know remarkably little about intellectual property laws more generally and report little experience as users or creators of creative works.

## Introduction

As of January 2017, over six million videos on YouTube contain the curious message: “no copyright intended” or “no copyright infringement intended.” Nearly as curious is that these videos routinely contain copyrighted material that the video poster does not have permission to use. Thus it appears that the video posters seem to believe that citing the original source of the copyrighted material, in combination with a statement that this poster is not the copyright holder, somehow reduces or eliminates the video poster’s culpability for copyright infringement. However, in the United States and most countries, this behavior is often illegal [1]. It appears that hundreds of thousands of YouTube posters have a different viewpoint about the most basic tenets of copyright law. In this paper, we explore the possibility that a large swath of the American public holds the beliefs that intellectual property protection is designed to prevent plagiarism and that acknowledging the source of creative work makes it legal for a subsequent user of that work to duplicate the material without permission.

Past work has used the term *plagiarism fallacy* to refer to a set of beliefs held by American adults that suggest they have major concerns with plagiarism. That is, they believe plagiarism concerns underlie U.S. IP protection and that remediation of IP infringement is possible through, amongst other things, attribution of credit [1]. These beliefs are considered to be a *fallacy* because lawmakers and legal experts do not consider plagiarism concerns to be a basis for intellectual property protection and IP laws generally do not permit use of others’ creative works simply by providing attribution [2–4]. We explore this plagiarism fallacy in the current work.

### The legal basis for IP protection

Intellectual property law in the United States is commonly understood by policymakers and legal experts to be designed primarily to incentivize the creation of novel, transformative products of the mind [2]. According to this logic, without potential protection, creators would not invest their time in the invention and development of new medicines, artistic work, or advancing technology. Of course, the justification for IP law is not singular, and alternate views have been advocated over time. For example, some commentators have focused on John Locke’s natural rights theory to argue that creators should hold *natural rights* in their intellectual creations or that by virtue of birthing a creation, the creator deserves the right to control the copying and distribution of their efforts [3]. The work of Kant and Hegel has been the basis for yet another argument, an *expressive rights* argument—the belief that providing intellectual property protection encourages cultural development and the expression of personal freedom, personality or beliefs [4]. Critically, however, recent empirical legal work suggests that the lay public may not agree with any of these arguments as the foundation for IP. Instead, the theory that appears most commonly to underlie lay views of IP protection is anti-plagiarism.

### Lay intuitions on plagiarism and attribution

In one study, Mandel, Fast, and Olson (see Study 2 [1]) presented 116 adults recruited through Amazon mechanical Turk with four possible justifications for IP protection—the three views widely identified by legal experts and scholars to actually underlie IP protection in the U.S. (incentives, natural rights, and expressive rights)—along with an alternative justification—to prevent plagiarism. Inconsistent with the actual law and viewpoints of IP law experts, but consistent with the *plagiarism fallacy*, plagiarism was the most widely endorsed reason for IP protection.

Likely because people view plagiarism as the primary legal concern in the re-use of creative works, they appear to believe that attribution to the original source of material reduces culpability for its re-use. Mandel and colleagues [1] first demonstrated this effect. They presented 443 mTurk participants with a series of vignettes in which one person (User) re-used, without permission, material first created by someone else (Creator). Participants were asked how permissible this re-use was. Then participants were told to assume that the User (still without permission) had credited the Creator as the original source and were asked how permissible the re-use was in this case. In every scenario—spanning material covered by copyright laws such as music and painting to material covered by patent laws such as vaccines and software—participants believed it *was* more permissible and *should be* more permissible to re-use the material when the original source was cited than when the original source was not cited. Further, in nearly every case, the majority of participants believed that attribution made it permissible for the User to make the copy if the User simply attributed the original creation to the Creator. The ameliorative effect of attribution was replicated in two additional studies using mTurk participants [5]. Consistent with these results, young children have been shown to believe that attribution to a source makes copying more permissible [6]. Yet, importantly, in the U.S., attribution does not make re-use any more legal. In fact, all of the scenarios presented to participants in the work of Mandel, Fast, and Olson [1] would constitute intellectual property infringement under U.S. IP law. Thus, past work suggests a very clear disconnect between the actual U.S. copyright and patent law and lay people’s intuitions about these laws.

### Limitations of past work

While early findings demonstrated the plagiarism fallacy, there are limitations to the past work. Chief amongst these limitations is the lack of representativeness of the samples utilized. All of these prior data were collected using Amazon mechanical Turk, an online marketplace of workers who complete surveys (and other tasks) in exchange for pay. While mTurk has proven to be a useful testing ground for behavioral research [7], the lack of diversity within this sample could be a major problem. As Paolacci and Chandler [8] have warned, findings from Amazon mechanical turk “should not be treated as representative of the general population” (p. 185).

The specific mTurk samples used in all of the previous plagiarism work have been disproportionately young, liberal, educated, and White, relative to the national population [1,5]. These demographic characteristics could explain all or part of the effects observed. For example, past research has found that liberals and people with greater education are less concerned about money [9,10], which could lead to a lower belief in financial incentives as motivation for creative work. In addition, greater education also means higher exposure to academic norms, which tend to utilize a plagiarism-based model in which people gain reputation via citations or attribution to their work. In this way, greater exposure to academic norms may in fact increase plagiarism concerns, drowning out other concerns. Alternatively, greater education could result is greater knowledge of the law (insofar as education is on topics related to law), which runs contrary to both the plagiarism fallacy and the attribution fallacy, thus actually decreasing the biases displayed in past work. While past work did not specifically observe a correlation between education and the plagiarism fallacy or attribution fallacy, perhaps the lack of a large enough sample of conservatives and participants with less education made it difficult to observe such an effect. Investigating the same phenomena within a more diverse and representative sample is crucial if one wants to draw conclusions about Americans overall, rather than just highly educated or liberal, young Americans. The current work aims to fill this gap in knowledge.

### Current work and hypotheses

This study was designed to ask whether past findings concerning the plagiarism fallacy replicate in a broader, representative sample of U.S. adults. Specifically we wanted to re-test the hypotheses that the lay public (1) believes that anti-plagiarism is the most important reason for IP law, and (2) believes that copying without permission should be more permissible if attribution to the original source of the creative work is made.

Further, for comparison to past work, we explored whether demographic differences, experience with IP, and/or knowledge about IP are associated with beliefs about plagiarism, attribution, and general views about IP protection. Previous work by Mandel and colleagues [1] as well as work by Fast and colleagues [5] found that knowledge of IP law is low and that most people report fairly limited experience with intellectual property (as either creator or user). In both papers individuals who knew more about the law were less likely to believe attribution should (or does) influence culpability when creative work is re-used by another person. Further, Mandel and colleagues [1] found that older, female, and more conservative individuals endorsed greater need for IP law compliance. Fast and colleagues [5] similarly found that older, female, and employed participants more strongly support IP protection in one study, and that older and female participants more strongly supported IP protection in a second study. We re-examine these relationships in a broader, more representative sample here.

## Method

### Participants

This research was approved by both the University of Washington’s Human Subjects Division and Temple University’s IRB. The sample included 506 adults (*M*_age_ = 43.67 years, *SD* = 14.70 years; 51% female) residing in the U.S. Participants were recruited in May 2016 through an online participant recruitment company that compensated participants for completing an online survey. The company was hired to recruit a sample that was demographically representative of the U.S. population as a whole. See Table 1 for detailed participant demographic information. The sample was selected to be representative of the national U.S. adult population across a variety of demographic characteristics, including gender, age, and race. Participation was completed online, thus participants were given informed consent via an information statement at the beginning of the online survey. Participants were required to agree to the information before proceeding with the survey.

**Table 1:** Demographics of participants.

Variable	Category	Percent of Sample
**Age**		
	18–29	17.7%
	30–39	33.8%
	40–59	30.2%
	60 and older	11.1%
**Gender**		
	Male	48%
	Female	51%
	Other	.2%
	Do not wish to report	.2%
**Race**		
	Non-Hispanic White or Euro-American	59%
	Black, Afro-Caribbean, or African American	19%
	Latino or Hispanic	10%
	Native American or Alaskan Native	2%
	South Asian or Indian American	5%
	Middle Eastern or Arab American	.2%
	Other or more than one	4%
**Education**		
	Less Than High School	1%
	Some High School	2%
	High School Grad	19%
	Some College	22%
	Trade/Technical Training	5%
	Associate Degree	12%
	Bachelor's Degree	27%
	Some Postgrad Work	2%
	Postgrad Degree	10%
**Residence**		
	Urban	26%
	Suburban	44%
	Small Town	14%
	Rural	16%
**Political Ideology**		
	Very Conservative	10%
	Conservative	19%
	Moderate	38%
	Liberal	17%
	Very Liberal	9%
	Other	.2%
	Don’t know	6%
**Employment**		
	Unemployed	14%
	Part-Time	14%
	Full-Time	50%
	Student	4%
	Retired	13%
	Other	5%
**Income**		
	Less than 10,000	7%
	10,000–19,000	7%
	20,000–29,000	12%
	30,000–39,000	12%
	40,000–49,000	10%
	50,000–74,000	21%
	75,000–99,000	14%
	100,000–150,000	8%
	More than 150,000	5%
	Do not wish to report	4%

### Materials and procedure

The survey had five phases: (1) an item ranking the participant’s preferred basis for IP law; (2) vignette evaluations; (3) general IP opinion items; (4) IP knowledge and experience questions; and (5) demographic information questions. Whether the ranking item came before or after the vignettes was counterbalanced but all other items were presented in the order listed above. The specific set of items used in this survey was not validated on a separate group of participants; however, all items were directly taken or adapted from previous work [1,5]. The online survey is in S1 Appendix.

#### Ranking potential bases of IP law

To assess the plagiarism fallacy, participants were presented with four potential reasons why there are laws regulating the products of creativity and innovation and asked to rank these four bases for IP law, ranging from what they thought is the most important (1) to what they thought is the least important (4). Each of the four justifications for IP law (incentives, natural rights, expressive rights, and anti-plagiarism) was described in one paragraph (see S1 Appendix for exact wording of this ranking item). This item was adapted from Mandel, Fast, & Olson [1].

#### Vignette evaluations

To assess the attribution fallacy, participants were presented with 8 vignettes reflecting a 2 (Intellectual Property Type: “expression” [copying some expression from a creative product into another work] and “product” [copying the complete creative product]) X 4 (Subject Matter: engineering, medical, music, and painting) design. The full vignettes are listed in the S1 Appendix. In each vignette, a person copied all or part of a product that was created by another person. Following each vignette, participants were asked “In your opinion, should [the property user’s] action be allowed?” Importantly, participants were asked to indicate their “personal opinion[s] about whether the action should or should not be allowed, regardless of what the law might actually be.”

Participants were then asked if the action should be permissible if the user had credited the original source of the property: “In your opinion, if [property user] gives [creator/property owner] credit as the source, should [property user’s] action be allowed?” Participants reported their answers to each question using a scale ranging from 1 (definitely not allowed) to 6 (definitely allowed). These questions were taken from previous work [1,5].

#### General IP opinion items

In order to measure participants’ level of support for intellectual property law more broadly, four general IP opinion questions were included in the survey (taken from [5]). The four questions were: 1) Do you think intellectual property laws in the United States should generally be made stronger, weaker, or left about where they are?; 2) How important do you believe it is for people to comply with intellectual property rights laws?; 3) How carefully do you comply with intellectual property laws?; and 4) Intellectual property laws should be most concerned with the rights of the: creator vs. user?. Participants responded to the questions using a slider scale ranging from 0 to 100, which was anchored at 50 when the item was presented to participants. For the first three questions, the lower end of the scale represented support for weaker IP protection and the higher end support for stronger IP protection (anchors: question 1, weaker…stronger; question 2, not important…important; and question 3, not carefully…carefully). For the fourth question, the lower end of the scale represented strong support for the creator of IP, whereas the higher end of the scale represented support for the consumer of IP (anchors to question 4: creator…user). This final item was reverse-scored in order for the scale to conceptually align with the other three items.

#### IP knowledge and experience questions

Participants were asked ten multiple choice questions about intellectual property law and two questions about their level of experience with IP creation or use (taken from [1,5], see those papers for full list of items). These items were included to assess their actual knowledge and training with regard to IP law. For example, in the knowledge quiz, participants were asked “What is permissible under copyright law, in general, concerning material found on the Internet: (a) It can be copied to other websites or downloaded freely; (b) It can be copied to other websites freely, but not downloaded; (c) It can be copied to other websites if attribution to the original site is provided; or (d) It can be copied to other websites if the author grants permission.” With regards to experience, participants responded to questions about their level of experience as a creator or producer of works protected by intellectual property and a user of intellectual property on five-point scales ranging from no experience (1) to considerable experience (5).

#### Demographic questions

The demographic section of the survey included questions about participant age, gender, race, education, employment status, income, political ideology, and type of residence. Responses to demographic questions are presented in Table 1.

## Results

### Ranking potential bases of IP law

Participants’ rankings of the possible bases for IP protection were not random, *χ*^*2*^(3) = 60.59, *p* < .001. Specifically, participants ranked Plagiarism (39%) as the most important reason for having IP laws more often than the other rationales (Natural rights: 24%; Incentives: 21%; Expressive rights: 16%). Expressive rights (36%) were most likely to be ranked least important compared to the other three bases (Incentives: 24%; Natural rights: 21%; Plagiarism: 19%), again with these answers differing from random chance, *χ*^*2*^(3) = 33.06, *p* < .001. See Table 2 for a full breakdown of participant rankings.

**Table 2:** Participant rankings of potential reasons for IP law.

	Ranked 1^st^	Ranked 2^nd^	Ranked 3^rd^	Ranked 4^th^
**Plagiarism**	39%	24%	18%	19%
**Natural Rights**	24%	32%	22%	21%
**Incentives**	21%	25%	30%	24%
**Expressive Rights**	16%	19%	30%	36%

### Vignette evaluations

Responses to the vignettes were analyzed in a 2 (Intellectual Property Type: expression vs. complete product) x 4 (Subject Matter: music vs. painting vs. engineering vs. medical) x 2 (Mitigating Factor: baseline vs. attribution) mixed measures ANOVA with Intellectual Property Type as a between-subjects factor and Subject Matter and Mitigating Factor as within-subjects factors (the S1 File provides additional information about the study design and analyses). Significant effects were followed-up with pairwise Bonferroni-corrected comparisons. The percent of participants responding that the actions should be allowed (by providing a response of 4, 5, or 6 on the scale) are presented in Table 3.

**Table 3:** Percentage of participants responding “allowed” to vignette subject matters.

	Baseline	Attribution
**Music**	49%	67%
**Painting**	58%	73%
**Medical**	48%	66%
**Engineering**	50%	62%

Percentage of participants who believed copying behaviors in the vignettes should be “allowed”. If a participant chose a 4, 5, or 6 on the 1 (definitely not allowed) to 6 (definitely allowed) scale, they were coded as saying the behavior should be “allowed”.

Analyses revealed a significant main effect of Intellectual Property Type on evaluations, *F*(1,499) = 62.90, *p* < .001, *η*_*p*_^*2*^ = .112, such that participants believed, overall, that copying the complete creative product (*M* = 3.36, *SD* = 1.13) should be less permissible than copying some, but not all, of the expression (*M* = 4.15, *SD* = 1.12). Analyses also revealed a significant main effect of Subject Matter on evaluations, *F*(3,1497) = 12.38, *p* < .001, *η*_*p*_^*2*^ = .024. Overall, participants found copying in the painting vignette (*M* = 4.02, *SD* = 1.39) less permissible than reusing material in the other three items (music: *M* = 3.66, *SD* = 1.54; engineering: *M* = 3.68, *SD* = 1.48; medical: *M* = 3.66, *SD* = 1.57), all *p*s < .001. Responses to the other three Subject Matters (music, engineering, and medical) did not differ from one another, all *p*s = 1.0. Finally, there was a significant main effect of Mitigating Factor on evaluations, *F*(1,499) = 158.94, *p* < .001, *η*_*p*_^*2*^ = .242, consistent with the Attribution fallacy, such that using someone else’s property without providing attribution to the original source (*M* = 3.46, *SD* = 1.25) was seen as less permissible than using someone else’s property while providing attribution to the original source (*M* = 4.05, *SD* = 1.21), *p* < .001.

These main effects were qualified by multiple interactions. Analyses revealed a significant interaction between Intellectual Property Type and Subject Matter, *F*(3,1497) = 20.05, *p* < .001, *η*_*p*_^*2*^ = .039. Participants saw it as less permissible to copy a complete product than the expression of a product within the painting, engineering, and medical vignettes, all *p*s < .001, but not for the music vignette, *p* = .256.

Further, analyses revealed a significant interaction between Subject Matter and Mitigating Factor, *F*(3,1497) = 3.98, *p* = .008, *η*_*p*_^*2*^ = .008. While in all cases participants thought it was more permissible to copy the creator’s work when attribution was given than when it was not, the degree of this difference between attribution and baseline differed by subject matter: music *M* = .672, engineering *M* = .685, painting *M* = .517 and medical *M* = .451.

Finally, there was a significant three-way interaction between Intellectual Property Type, Subject Matter and Mitigating Factor, *F*(3,1497) = 6.79, *p* < .001, *η*_*p*_^*2*^ = .013. Specifically, attribution increased permissibility compared to the baseline condition more in the expression condition compared to the physical product condition for music vignette (mean differences = .835 and .510, respectively); in contrast, attribution increased permissibility compared to the baseline condition more in the complete product condition compared to the expression condition for the painting (mean differences = .629 and .405, respectively), engineering (mean differences = .792 and .579, respectively) and medical (mean differences = .629 and .273, respectively) vignettes. That is, although there was an attribution fallacy for all vignettes, the degree of that effect differed depending on Domain and Property Type.

### General IP opinion items

Participants’ responses to all four of the general IP opinion questions were significantly higher than the midpoint of the scale (50), suggesting that participants tended to: (1) favor stronger IP laws, *t*(498) = 22.65, *p* < .001; (2) support compliance with IP laws, *t*(498) = 36.30, *p* < .001; (3) report self-compliance with IP laws, *t*(496) = 30.41, *p* < .001; and (4) support laws that are concerned with the rights of creators of IP, *t*(496) = 4.97, *p* < .001. Means and standard deviations for each of the general IP opinion items are listed in Table 4. Participants’ scores on the first three opinion questions were highly related to one another (Cronbach’s alpha = .74); therefore, we computed a factor called *IP Support*, which is the average of scores on these three items. Higher IP Support scores indicate greater support for the strength of and compliance with IP law. This IP Support factor was used to assess individual difference analyses below. The fourth item reduced the reliability of the composite and therefore was excluded from subsequent analyses.

**Table 4:** Means of general IP opinion questions.

	M (SD)
**IP laws in the USA should be stronger, weaker, or the same?**	69.66 (19.39)
**How important is it to comply with IP laws?**	81.06 (19.11)
**How carefully do you comply with IP laws?**	79.60 (21.70)
**Laws should be concerned with rights of: creator vs. user**	57.76 (34.78)

### IP knowledge and IP experience

An IP Knowledge score was calculated for each participant, representing the number of correct responses on the 10-item intellectual property quiz (*M* = 3.29, *SD* = 1.57). Participants tended to perform better than chance (2.5 out of 10 correct) on the IP knowledge quiz, *t*(501) = 11.22, *p* < .001, though performance was still poor (well below 50%), consistent with the findings reported Mandel et al [1].

Participants reported having little to no experience as a creator or producer of IP (*M* = 1.80, *SD* = 1.18) or as a user of IP (*M* = 2.00, *SD* = 1.34), again consistent with Mandel et al [1]. These items were significantly positively correlated, *r* = 0.73, *p* < .001, thus we averaged these items to create an IP Experience score for each participant.

### Individual difference analyses

We examined the correlations between individual difference measures and the main dependent measures. Importantly, because of the large number of correlations conducted, which inflates Type I error rates, we assessed significance of correlations with a p-value cutoff of *p* < .01. We first paid specific attention to whether the previously reported findings [1,5] replicated. Unlike in both previous papers, we found that IP knowledge was not significantly associated with responses to the attribution items. That is, people who knew more about IP as demonstrated by our quiz were no more or less likely to believe that attribution should affect the legal permissibility of copying. In addition, while past work [1,5] found that older, female, more conservative and employed participants more strongly support IP protection, in this more diverse, representative sample, none of the demographic variables was significantly correlated with IP support.

## Discussion

Overall, we found, in a large replication study, that previous findings reporting the plagiarism fallacy are robust in a more diverse and representative sample. Specifically, many Americans believe that the most important reason why IP rights exist is to prevent plagiarism, rather than to create incentives for creative work, to promote expressive rights, or to honor natural rights. Further, and likely as a result of this concern for plagiarism, adults believe that acknowledging the source of creative work should make copying that work more permissible, regardless of actual permission. In fact, the majority of participants in the majority of scenarios believed that copying others’ work (without permission) should be permissible as long as the user attributed the source. These findings suggest that past work with demographically-limited samples [1,5] are not only replicable but replicable in more diverse samples. In recent years, a push to replicate major findings and to assess their generalizability has been advocated across fields of science [11–14].

In addition to the primary findings, we examined several other findings from the past literature. Two additional findings that replicated were the observation that, in general, the lay public knows very little about intellectual property law and that the lay public feels that they have limited experience as users or creators of creative products. We also re-investigated the relationship between our primary dependent variables and a range of demographic variables. None of the primary individual difference relationships replicated. Thus, we believe that the main effects—the plagiarism fallacy and the attribution fallacy—are more robust and do not appear to rely on the representativeness (or lack thereof) of the sample. The individual differences however, appeared less reliable across studies with different demographic compositions.

Although the current work had a much more diverse sample of participants compared to past work, the current study only included participants who reside in the United States. Thus one limitation of this work is that it reflects the views of Western, educated, industrialized, rich, and democratic (WEIRD) individuals, whose opinions likely do not generalize to non-WEIRD individuals [15]. Future work could explore the tendency to endorse the plagiarism fallacy across cultures. Another potential limitation of the current study is that the vignettes were directly drawn from previous studies, making it difficult to know whether participants’ consistent responses to the vignettes from past to current work depend on those particular scenarios, or whether their responses would generalize to other scenarios and even other domains of infringement. Therefore, future work could investigate individuals’ responses to copying in new scenarios and domains.

Another limitation in our study design was our use of the ranking item for assessing people’s beliefs about the basis of IP protection. Specifically, we provided four possible reasons, but participants may have had other ideas not represented by these four possibilities. Thus, we can only conclude that plagiarism was the most common amongst these four options rather than that plagiarism would necessarily have been the most endorsed in a range of other options. We are skeptical that this is a major limitation, however, because Mandel and colleagues [1] did ask a more open-ended question and found that participants frequently indicated concerns about plagiarism as the reason for concerns in infringement cases. Further, our findings that attribution made copying more permissible suggest that plagiarism concerns are important to participants. However, future work might address this concern with more open-ended responses.

Despite these limitations, our replication of the lay public’s beliefs concerning attribution is particularly notable, as all of the scenarios in this study were specifically selected because these actions constitute intellectual property rights infringement under current U.S. IP law. While about half of respondents endorsed behavior that violates IP law on the baseline item, respondents were significantly more likely to do so on the attribution item. That is, the majority of respondents on most of the vignettes believed that providing attribution to the original source should make copying behavior permissible, even though the law and lawmakers do not permit such behavior. Such a finding is critical for legal scholars and policy-makers to understand. Where individuals disagree with a law, they are less likely to view the law as legitimate and less likely to comply with the law [16].

The attribution finding is also worthy of note in light of recent findings about creators’ preferences. Sprigman, Buccafusco, and Burns [17] found that creators themselves appeared to value attribution. In their study, creators were willing to accept less pay in exchange for attribution of their work. Thus it appears that not only third party observers, with little experience in creating these works, like the participants in the current work, but also actual creators, value attribution in a way that U.S. law does not. Intriguingly, many European countries provide for certain attribution rights for authors under their copyright laws [18]. This system may in fact better align with the way American adults intuitively reason about creative works.

For legal systems to work, people governed by that system need to know, understand, and follow that governance. The current work suggests that beliefs about intellectual property protection that differ from those of legal experts extend beyond those individuals who create videos on YouTube or participate in behavioral experiments on Amazon mechanical Turk, and instead are fairly representative of the American public. Such findings should be discomforting to policy-makers and suggest the need for greater education and intervention, or changes in legal policy.

## Supporting information

S1 Appendix Online survey. (DOCX)

S1 File Additional study design & analysis information. (DOCX)
