# Supplementary material for: Intuitive intellectual property law: A nationally-representative test of the plagiarism fallacy
Source: PLoS One. 2017 Sep 1;12(9):e0184315. doi: 10.1371/journal.pone.0184315 (PMC5581163; doi:10.1371/journal.pone.0184315)
Supplement: S1 Appendix — (DOCX) [file pone.0184315.s001.docx]

**S1 Appendix**

**Online Survey**

Ranking question about basis for IP law

Instructions: Each paragraph below describes a reason why there are laws regulating the products of creativity and innovation. Using your cursor, drag the paragraphs into a ranked order that represents the most important reason for legally protecting the intellectual property to the least important reason for legally protecting intellectual property. Position number 1 should designate the reason that you think is the most important basis for intellectual property law, whereas position 4 should designate the reason that you think is the least important basis for intellectual property law.

We have intellectual property laws because we value the development and production of creative works and innovative products. These laws serve as a means of **encouraging** creation and innovation by allowing people to profit off of their creations and inventions. Providing an opportunity to profit for creators and innovators with intellectual property laws produces an **incentive** for people to create and innovate.

We have intellectual property laws because we value the insight and effort required to achieve creative works and innovative products. These laws serve as a means of protecting people’s inherent, natural rights they are **entitled**to in their creations and inventions. Providing protection for creators and innovators with intellectual property laws protects people’s **inherent rights** in their creations and inventions.

We have intellectual property laws because we value the ability to express and distinguish ourselves in creative works and innovative products. These laws serve as a means of allowing and enabling people to **express**themselves in their creations and inventions. Valuing the opportunity to express oneself with intellectual property laws protects people’s ability to express their **identity**creatively and through innovation.

We have intellectual property laws because we value creative works and innovative products. These laws serve as a means of preventing people from **plagiarizing** another person’s creation or invention. Protecting creators and innovators with intellectual property laws prevents people from **claiming**another person’s creations or inventions as their own.

Permissibility of Copying Scenarios

Instructions: You will now answer questions about four different scenarios. Each scenario has two questions, with one question appearing per page along with the scenario. The questions will ask you whether something **should or should not be allowed**. In these questions we are interested in **your personal opinion**about whether the action should or should not be allowed, regardless of what the law might actually be.

***Music Vignettes***

*Expression Condition*

The classic fusion band, Garage Feet, wrote, recorded, and copyrighted the ballad “Don’t Stomp on My Heart,” which topped music charts in the summer of 1990. The song, written in the key of A minor, is considered by many music scholars to be the first known track to blend upbeat reggae and jazz instrumentals. An admirer of Garage Feet named Bill records a different version of “Don’t Stomp on My Heart,” played in B minor and at half speed.  Bill changes some of the lyrics from “Don’t Stomp on My Heart,” while keeping most of the original chorus.

*Complete Product Condition*

The classic fusion band, Garage Feet, wrote, recorded, and copyrighted the ballad “Don’t Stomp on My Heart,” which topped music charts in the summer of 1990. The song, written in the key of A minor, is considered by many music scholars to be the first known track to blend upbeat reggae and jazz instrumentals. An admirer of Garage Feet named Bill purchases a digital version of “Don’t Stomp on My Heart,” and likes it so much that he figures out how to extract the song into an mp3 file. Bill adds a short introduction at the beginning of the song and then emails the mp3 to several of his friends.

Baseline Item: In your opinion, should Bill’s action be allowed?

1 (definitely not allowed) to 6 (definitely allowed)

Attribution Item: In your opinion, if Bill gives Garage Feet credit as the source, should Bill’s action be allowed?

1 (definitely not allowed) to 6 (definitely allowed)

***Painting Vignettes***

*Expression Condition*

Charles O’Malley, an Irish artist trained in collage design, gained fame after developing a newspaper based collage technique. O’Malley premiered his newspaper collage style in his piece titled Spotlight, which depicts a view from the top of the famous Cliffs of Moher in Ireland. After traveling to Ireland to study Charles O’Malley's artwork and view Spotlight in person, an aspiring artist named Randall decides to create his own interpretation of Spotlight. Using oil paints on a similarly sized canvas, Randall paints a picture of the Spotlight collage.

*Complete Product Condition*

Charles O’Malley, an Irish artist trained in collage design, gained fame after developing a newspaper based collage technique. O’Malley premiered his newspaper collage style in his piece titled Spotlight, which depicts a view from the top of the famous Cliffs of Moher in Ireland. After traveling to Ireland to study Charles O’Malley and view Spotlight in person, an aspiring artist named Randall decides to run a computer analysis of the artwork in order to determine the exact paper, collage glue, and angles of lines used in the art, and makes several painstaking replications of Spotlight.

Baseline Item: In your opinion, should Randall’s action be allowed?

1 (definitely not allowed) to 6 (definitely allowed)

Attribution Item: In your opinion, if Randall gives Charles O’Malley credit as the source, should Randall’s action be allowed?

1 (definitely not allowed) to 6 (definitely allowed)

***Engineering Vignettes***

*Expression Condition*

Several years back, an electrical engineer named Gary, realized that automobiles could be designed to drive themselves.  Gary developed a new semiconductor chip that could be installed in most automobiles to independently drive and navigate the vehicle.  The semiconductor processes information about road conditions including traffic and road signs.  Gary obtained a patent on his semiconductor device.  After learning of Gary’s invention, a fellow electrical engineer named Milton reverse engineered it and programed a similar automobile semiconductor chip that will not only navigate the vehicle and process road conditions but also avoid hazards in the roadway and park itself.

*Complete Product Condition*

Several years back, a electrical engineer named Gary, realized that automobiles could be programmed to drive themselves.  Gary developed a new semiconductor chip that could be installed in most automobiles to independently drive and navigate the vehicle.  The semiconductor processes information about road conditions including traffic and road signs.  Gary obtained a patent on his semiconductor device.  After learning of Gary’s invention, a fellow electrical engineer named Milton designed a semiconductor chip manufacturing device to make semiconductor chips that are replicas of Gary’s.

Baseline Item: In your opinion, should Milton’s action be allowed?

1 (definitely not allowed) to 6 (definitely allowed)

Attribution Item: In your opinion, if Milton gives Gary credit as the source, should Milton’s action be allowed?

1 (definitely not allowed) to 6 (definitely allowed)

***Medical Vignettes***

*Expression Condition*

HealthCorps, a pharmaceutical company, comes up with the idea that it might be possible to protect against West Nile Flu, a disease previously thought to be incurable, with a vaccine.  HealthCorps develops and obtains a patent on the chemical structure of a West Nile Flu vaccine.  A competing company, Everlife, notices the success of HealthCorps's vaccine, and figures out how to use a different manufacturing process to produce a vaccine with the same chemical structure as HealthCorps's.

*Complete Product Condition*

HealthCorps*,*a pharmaceutical company, comes up with the idea that it might be possible to protect against West Nile Flu, a disease previously thought to be incurable, with a vaccine.  HealthCorps develops and obtains a patent on the chemical structure of a West Nile Flu vaccine.  A competing company, Everlife, notices the success of HealthCorps's vaccine, purchases one of the vaccines manufactured by HealthCorps, and does a chemical analysis to develop and manufacture duplicate copies of HealthCorps’s.

Baseline Item: In your opinion, should Everlife’s action be allowed?

1 (definitely not allowed) to 6 (definitely allowed)

Attribution Item: In your opinion, if Everlife gives HealthCorps credit as the source, should Everlife’s action be allowed?

1 (definitely not allowed) to 6 (definitely allowed)

Part 3: General IP Support Questions

1. Do you think Intellectual Property laws in the United States should generally be made stronger, weaker, or left about where they are?

0 (weaker) to 100 (stronger)

2. How important do you believe it is for people to comply with intellectual property rights laws?

0 (not important) to 100 (important)

3. How carefully do you comply with intellectual property laws?

0 (not carefully) to 100 (carefully)

4. Intellectual property laws should be most concerned with the rights of the:

0 (creator) to 100 (user)

Part 4: IP Knowledge Questions [correct answers are bolded]

1. Which of the following statements most accurately describe the similarities and differences between the patent system and the copyright system?

1. **Patent law and copyright law cover different types of creative works, and the procedure for obtaining a patent operates very differently from the procedure for obtaining a copyright**
2. Patent law and copyright law cover different types of creative works, yet the procedure for obtaining a patent operates similarly to the procedure for obtaining a copyright
3. Patent law and copyright law cover similar types of creative works, yet the procedure for obtaining a patent operates very differently from the procedure for obtaining a copyright
4. Patent law and copyright law cover similar types of creative works, and the procedure for obtaining a patent operates similarly to the procedure for obtaining a copyright

2. A patent could cover, in general:

1. Any innovative idea
2. Any creative work
3. Any innovative discovery of scientific phenomena

**(d) Any innovative tangible product**

3. To obtain a copyright, someone must:

1. File the copyright material with the U.S. Copyright Office
2. File the copyright material and obtain copyright approval from the U.S. Copyright Office
3. Mail the copyright material to themselves in a sealed envelope

**(d) Do nothing particular with the copyright material**

4. Which of the following most accurately describes the rights provided by a patent on an invention to restrict the actions others can legally take?

1. Others can still make the invention for non-commercial use and can resell a copy of the invention that they legally purchased
2. **Others cannot make the invention for non-commercial use; however others can resell a copy of the invention that they legally purchased**
3. Others cannot resell a copy of the invention that they legally purchased; however others can make the invention for non-commercial use
4. Others cannot make the invention for non-commercial use and cannot resell a copy of the invention that they legally purchased

5. Which of the following statements best summarizes the fair use exception to copyright protection:

1. A person may copy copyrighted material when only a limited portion of the work is copied
2. A person may copy copyrighted material when it is used for educational purposes
3. A person may copy copyrighted material when it is not used for financial gain
4. **A person may copy copyrighted material in limited cases based on the type of work and effect of the use on sales**

6. Copyright protection can cover, in general:

1. Any creative idea
2. Written works, but not other forms of creative work
3. **Creative work that is written or recorded in some tangible form**
4. Any creative activity

7. How long does the standard patent protection term last?

1. **20 years**
2. 70 years
3. The life of the creator plus 20 years
4. The life of the creator plus 70 years

8. Someone comes up with an original achievement and obtains intellectual property protection for it. Sometime later, a second person comes up with a nearly identical achievement, completely independently and without any knowledge of the earlier work. Which kind of intellectual property right might the second person be able to obtain?

1. **A copyright (but not a patent)**
2. A patent (but not a copyright)
3. Either a copyright or a patent
4. Neither a copyright nor a patent

9. To obtain a patent, someone must:

1. File the patent material with the U.S. Patent Office
2. **File the patent material and obtain patent approval from the U.S. Patent Office**
3. Mail the patent material to themselves in a sealed envelope
4. Do nothing particular with the patent material

10. What is permissible under copyright law, in general, concerning material found on the Internet:

1. It can be copied to other websites or downloaded freely
2. It can be copied to other websites freely, but not downloaded
3. It can be copied to other websites if attribution to the original site is provided

**(d) It can be copied to other websites if the author grants permission**

Part 5: IP Experience Questions

1. Do you have any experience as a **creator or producer** of works or products protected by intellectual property rights?

1 (no experience) to 5 (considerable experience)

2. Do you have any experience as a **user** of works or products created by others that are protected by intellectual property?

1 (no experience) to 5 (considerable experience)

Part 6: Demographics

***Age***

What is your age in years?

***Gender***

Which of these describes you most accurately?

Male; Female; Other; Do not wish to report

***Race***

Which of the following best represents your racial or ethnic heritage? Choose all that apply.

Non-Hispanic White or Euro-American

Black, Afro-Caribbean, or African American

Latino or Hispanic American

South Asian or Indian American

Middle Eastern or Arab American

Native American or Alaska Native

Other, please specify: ___________

***Income***

What is your estimated total annual household income?

Less than $10,000

$10,000 to $19,999

$20,000 to $29,999

$30,000 to $39,999

$40,000 to $49,999

$50,000 to $74,999

$75,000 to $99,999

$100,000 to $149,999

$150,000 or more

Do not wish to report

***Political Ideology***

Generally speaking, do you consider yourself to be:

Very conservative

Conservative

Moderate

Liberal

Very Liberal

Don’t know

Other, please specify: ___________

***Education***

What is the highest level of education you have completed?

Less than high school

Some high school

High school graduate (includes equivalency)

Some college

Trade/technical/vocational training

Associate Degree

Bachelor’s Degree

Some postgraduate work

Post graduate degree

***Employment***

How would you describe your employment status?

Employed full time

Employed part time

Unemployed

Student

Retired

Other, please specify: __________

***Residence***

In what kind of area do you reside?

Rural

Small Town

Suburban

Urban
