# Supplementary material for: Intuitive intellectual property law: A nationally-representative test of the plagiarism fallacy
Source: PLoS One. 2017 Sep 1;12(9):e0184315. doi: 10.1371/journal.pone.0184315 (PMC5581163; doi:10.1371/journal.pone.0184315)
Supplement: S1 File — (DOCX) [file pone.0184315.s002.docx]

**S1 File**

**Additional Study Design & Analysis Information**

**Study Design**

We also manipulated when participants completed the ranking item in the survey, such that half of the participants responded to the ranking item before the vignettes and half responded to the ranking item after the vignettes. An initial analysis was conducted including this additional between-subjects factor. This analyses revealed that the position of the ranking item in the survey only interacted with the Mitigating Factor item, *F*(1,497) = 5.10, *p* = .024, *η_p_^2^*= .010, such that the difference between the Baseline and Attribution items was significant in both conditions; however, this effect was smaller when participants completed the ranking item last compared with when they completed the ranking item first. Since this factor did not have a meaningful impact on participants’ responses (i.e., the effect did not go away or change directions) and was not a factor of interest, we eliminated this ordering factor from subsequent analyses for ease of comprehension and because there was no theoretical reason to predict this factor mattered.

**Individual Difference Analyses**

Because the IP Support composite was used in Fast, Olson, & Mandel (2016), and because it reduced the overall false positive rate, we utilized the composite here as well. However in Mandel, Fast, & Olson (2016) analyses were by item. Because of this, we re-ran the correlations involving the IP support composite for each item separately assessing whether we replicated any previous findings from Mandel, Fast, & Olson (2016). We did find that women, *r* = .199, *p* < .001, and older people, *r* = .159, *p* <.001 were more likely to indicate that they personally comply with IP law.
